# Efficacy of continuous epidural anesthetics and steroids infusion for management of acute herpes zoster and postherpetic neuralgia: a retrospective study

**DOI:** 10.3389/fneur.2025.1600592

**Published:** 2025-06-09

**Authors:** Xinyu Dou, Xinghai Guan, Chen Li, Wangjun Qin, Yi Zhang, Yifan Li, Bingyu Han, Chenglong Wu, Yangyang Chen, Li Wang, Peng Mao, Ling Zhou, Bifa Fan

**Affiliations:** ^1^Department of Pain Medicine, China-Japan Friendship Hospital, Beijing, China; ^2^Department of Pain Medicine, Wuhan Fourth Hospital, Hubei, China; ^3^Graduate School, Beijing University of Chinese Medicine, China; ^4^Department of Pharmacy, China-Japan Friendship Hospital, Beijing, China

**Keywords:** herpes zoster, postherpetic neuralgia, epidural infusion, anesthetics, steroids, pain management

## Abstract

**Background:**

Herpes zoster (HZ), caused by the reactivation of the varicella-zoster virus, frequently leads to postherpetic neuralgia (PHN), a debilitating neuropathic pain condition. Current treatments for acute HZ and PHN prevention remain suboptimal. This study evaluates the efficacy of continuous epidural infusion of anesthetics and steroids combined with conventional oral medication management (epidural infusion group) vs. conventional oral medication management (contrast group) alone in managing acute HZ and reducing PHN incidence.

**Methods:**

A retrospective analysis included 173 acute HZ patients [numerical rating scale (NRS) score ≥4] treated with epidural infusion group (*n* = 89) or contrast group (acyclovir, mecobalamin, and vitamin B1; *n* = 84). Epidural infusion group combined lidocaine (0.25%−0.5%, 0.5 ml/h) and betamethasone (0.3 ml/day) administered via epidural catheter for 3 days. Outcomes assessed skin lesion recovery, pain relief (NRS scores), PHN incidence, complications, and patient satisfaction over 3 months.

**Results:**

Epidural infusion group significantly improved skin lesion recovery (88.43 vs. 79.33% at 1 month, *P* < 0.001) and rash elimination (98.76 vs. 96.67% at 1 month, *P* = 0.039). Pain scores were lower in the epidural infusion group at all follow-ups (3 days to 3 months, *P* < 0.05). PHN incidence at 3 months was reduced with epidural infusion group (11.2 vs. 23.8%, *P* = 0.028), with higher complete remission rates (82.0 vs. 61.9%, *P* = 0.003). Complication rates were comparable between groups (*P* > 0.05), and patient satisfaction scores favored epidural infusion group (3.68 ± 1.01 vs. 4.18 ± 0.83, *P* < 0.001).

**Conclusion:**

Epidural infusion group demonstrates superior efficacy in alleviating acute HZ symptoms, accelerating skin healing, and reducing PHN risk compared to oral therapy, with comparable safety and higher patient satisfaction. This approach offers a promising strategy for HZ management, warranting further validation through large-scale prospective trials.

## 1 Introduction

Herpes zoster (HZ) is a viral disease characterized by a painful vesicular rash involving one or more adjacent dermatomes (1). HZ is caused by the reactivation of latent varicella zoster virus (VZV), which persists and hibernates in the dorsal root ganglion (DRG) after the initial infection. The reactivation of VZV leads to irritation in the nerve distribution area, abnormal sensitization of nociceptors, and hyperactivity of the central nerve system (2). VZV reactivation often occurs in patients with low cell-mediated immunity due to aging or immunosuppression (3, 4). The acute phase of HZ is currently defined as the period within 30 days of rash onset. During this period, the impaired skin area may be extremely painful, and it generally takes 14−21 days for the deflorescence, healing of the skin lesions and pain resolution (3, 5). If the patient is not treated timely and appropriately or suffers hypoimmunity in the acute phase, the residual pain could beyond the pathological recovery stage, resulting in postherpetic neuralgia (PHN).

As the most common complication of acute HZ, PHN is considered as a neuropathic condition characterized by refractory pain, and it is the consequence of inflammatory nerve damage secondary to VZV reactivation, which destroys the affected central and peripheral nerves and leads them to undergo inflammation and an immune responses. The incidence of PHN in HZ patients varies from 9% to more than 50% (3, 6). Severe physical, psychological, social, and functional disturbances as consequences of chronic refractory pain, resulting in a severe reduction in their quality of life. Therefore, patients at risk of developing PHN should to be treated aggressively with appropriate therapies. Advanced age, severe rashes, severe pain, prodrome, and severe pain with HZ are considered risk factors for PHN (7, 8). This shows that in order to reduce the occurrence and development of PHN, the timely intervention in the acute HZ is particularly critical. Currently, various therapies have been proposed for HZ intervention and PHN prevention, including antiviral agents, steroids, vaccines, anesthetics, anticonvulsants, antidepressants etc., through oral, single, or multiple epidural injections, and epidural block drug-delivery route (9). However, owing to the complex pathophysiology of PHN, despite the existence of many treatments, PHN remains difficult to manage despite the existence of many strategies (10, 11).

Continuous epidural infusion of anesthetics and steroids has been proposed to mitigate the inflammatory response and nociceptive input associated with HZ, potentially hindering the progression to PHN. Epidural administration of local anesthetics like lidocaine offers targeted pain relief by blocking nerve conduction at the spinal level, leading to sustained analgesia, provided significant pain relief and improved quality of life in patients with HZ during the first 3 months after rash onset (12). Steroids such as betamethasone, when administered epidurally, exhibit potent anti-inflammatory properties, reducing nerve inflammation and associated pain. Combining anesthetics and steroids in epidural infusions theoretically alleviates the inflammatory response and the accumulation of nociceptive input that might aggravate HZ and PHN, possibly retarding HZ development. Previous studies have reported the effectiveness of epidural injections or infusion of local anesthetics and steroids to control zoster-associated pain (ZAP) (13, 14). Despite these findings, there remains a paucity of research specifically evaluating the combined continuous epidural infusion of anesthetics and steroids, in the context of acute HZ treatment. Most existing studies have primarily focused on pain relief, often overlooking other therapeutic effects on HZ. Therefore, further research is warranted to comprehensively assess the potential benefits of epidural anesthetics and steroids infusion, including its impact on skin lesion healing, PHN prevention, and overall patient satisfaction.

In this study, a retrospective controlled trial was conducted to compare the efficacy and safety of continuous epidural anesthetics and steroids infusion with conventional oral medication management in the treatment of acute HZ. The therapeutic effects of epidural anesthetics and steroids infusion for acute HZ was comprehensively evaluated, rather than focusing solely on pain relief. Meanwhile, epidural anesthetics and steroids infusion was demonstrated to be effective in reducing the incidence of PHN.

## 2 Materials and methods

### 2.1 Study design and participants

Permission to conduct this study was obtained from the Institutional Ethics Committee of Wuhan Fourth Hospital, China (KY2024-197-01). The medical records of patients who underwent continuous epidural anesthetics and steroids infusion or conventional oral medication management for acute HZ from June 2022 to June 2024 were retrospectively analyzed and only included patients admitted to our pain clinic for ZAP rated as ≥4 on a numerical rating scale (NRS). Generally, hospital admission is usually recommended in patients with acute HZ with obvious symptoms. These patients were included in this study: epidural anesthetics and steroids were performed at the patient's own request in cases of refractory pain after the failure of oral gabapentin or pregabalin. Patients were excluded if they met any of these criteria: (1) previous epidural injection or infusion treatment for HZ within 6 months; (2) present immunosuppressive status; (3) hemostatic disorders or receiving antiplatelet therapy; (4) liver function damage or liver failure; (5) kidney function damage or kidney failure; (6) past or present conditions that may influence study participation, safety, or interpretation of the study results, such as cancer, severe diabetes, heart diseases, neurological disorders, dermatoses, or psychosis; and (7) not an appropriate study participant, according to the opinion of the investigators. In addition, to assess the efficacy and safety of continuous epidural infusion for preventing PHN in HZ acute phase, the cases with treatment initiation time longer than 1 month after the onset of HZ were excluded from this current study.

### 2.2 Procedure

1) Conventional oral medication management: contrast group

Patients in the contrast group received a standard treatment only, namely conventional oral medication management: mecobalamin 0.5 mg per dose, three times daily; acyclovir 0.2–0.8 g per dose, every 4 h, five times daily; vitamin B1 5 mg, three times daily.

2) Continuous epidural anesthetics and steroids infusion: epidural infusion group

Patients in the epidural infusion group received epidural anesthetics and steroids infusion therapy in addition to conventional oral medication management. Patients were placed in the prone position on the procedure table, and an aseptic dressing was applied to the procedure site after full disinfection. Firstly, under the guidance of C arm X-ray fluoroscopy, an 18G Tuohy needle was inserted into the interlaminar epidural space at three levels below the target level (the target level was confirmed based on the affected dermatomal distribution of rash and pain in advance). Subsequently, the loss of resistance technique was used to identify whether the Tuohy needle was accurately placed in the epidural space. After confirming the above result, a 20G epidural catheter was inserted through the Tuohy needle into the epidural space, and contrast agent was injected to identify placement of the catheter tip in the suitable target position. Finally, continuous epidural infusion of lidocaine (0.25% in the cervical segment; 0.5% in the thoracolumbar segment) at a rate of 0.5 ml/h was administered along with daily epidural infusion of betamethasone (0.3 ml/day) for 3 days via the epidural catheter and the AutoFuser pump, which is a portable balloon infusion device.

### 2.3 Data collection

Data retrieved and analyzed from the medical records included: sex, age, localization of herpes, duration of rash (time between HZ onset and first treatment), initial area of skin lesions, pain duration (time between pain onset and first treatment), initial pain severity (pain rating at first visit, baseline), and the pain rating at 3 days, 1 week, 1 month, and 3 months after the treatments. Patients reported the severity of their pain by rating it on the NRS (0 = no pain; 10 = unbearable pain). In addition, the degree of skin lesion recovery (defined as the percentage of skin lesion area reduced from HZ onset) and the elimination of rash numbers (defined the percentage of rash numbers decreased from HZ onset) at 1 week and 1 month after treatment were also collected. Furthermore, the number and details of side effects throughout the follow-up period were also recorded. At the end of follow-up (3 months after treatment), the information of patient satisfaction was recorded using 5-point Likert scale scores (1 = significantly aggravated; 2 = slightly aggravated; 3 = no change; 4 = slightly improved; 5 = significantly improved).

### 2.4 Outcome measures

The degree of skin lesions recovery and herpes elimination at 1 week and 1 month after treatment were compared to assess the efficacy of the two therapies for acute HZ. The baseline NRS pain scores of both groups were compared. To evaluate analgesic effects, the study compared the pain scores of the two groups at baseline and after 3 days, 1 week, 1 month, and 3 months after treatment. Given that the definition of PHN may vary across different studies or clinical guidelines, for example, some definitions specify that postherpetic neuralgia is only considered if pain persists for more than 3 months after shingles, while others set the threshold at more than 1 month, to determine the effect of the therapies on the incidence of PHN, the pain rating at 1 and 3 months after treatment was compared with the initial pain rating. Effective remission was considered as a ≥50% reduction in pain severity since the initial visit. Complete remission was defined as patients whose NRS pain score was ≤ 1 and who no longer needed medical support. The percentage of patients who achieved effective remission and complete remission and in each group were compared.

### 2.5 Statistical analysis

Data are presented as mean ± standard deviations (SD) for continuous variables. Kolmogorov–Smirnov test was used to evaluate the normality of demographic data. Comparisons between the groups were made using an independent *t*-test for normally distributed variables, and non-normally distributed variables were compared using the Mann–Whitney *U* test. The presence of pain (at the different time points) in the two groups was compared by calculating the odds ratio (OR) with 95% CI and tested with the χ^2^ test. A repeated measures analysis of variance with the Bonferroni *post-hoc* test was used to determine whether the pain score was significantly reduced at each time point after treatment compared to that at the baseline within each treatment group, and the differences in pain scores between the two groups using covariance analysis. Data were reported as mean ± standard deviation or median (inter-quartile range) and were analyzed with the Statistical Package for the SPSS (version 17.0, IBM, Chicago, IL, USA). All statistical tests were two-tailed, and the threshold for statistical significance was set at *P* < 0.05.

## 3 Results

At first, the study reviewed a total of 191 patients. Eighteen patients were excluded due to lack of complete follow-up medical information or did not meet the inclusion criteria. Finally, the medical records of 173 acute HZ patients were screened and collected, with 89 patients in epidural infusion group and 84 patients in contrast group.

Patient general and clinical characteristics are summarized in Table 1. Between the two groups, there were no statistically significant differences in baseline demographics and clinical characteristics, including sex, ages, duration of rash, initial area of skin lesions, pain duration, and initial pain severity (*P* > 0.05).

**Table 1:** Patient characteristics.

**Variable**	**Contrast group (*n* = 84)**	**Epidural infusion group (*n* = 89)**	***P*-value**
**Sex**			0.883
Male	34	37	
Female	50	52	
**Age (year)**			0.982
≤ 49	19 (37.37, 23–48)	6 (36.50, 25–43)	
50–79	48 (67.75, 54–79)	77 (65.56, 50–79)	
≥80	17 (87.06, 80–96)	6 (83.67, 80–89)	
**Pain**			
Severity (NRS score)	6.13 ± 1.89	6.29 ± 1.28	0.515
Duration (days)	20.02 ± 9.00	18.5 ± 8.47	0.254
**Localization**			/
Cervical	36	19	
Thoracic	40	50	
Lumbar	8	20	
Duration of rash	13.25 ± 6.60	12.15 ± 6.36	0.264
**Area of skin lesion (cm** ^2^ **)**			0.439
< 20	23	21	
20–50	27	37	
>50	34	31	

P-value, control group compared to the epidural infusion group.

To explore the efficacy of the two strategies for acute HZ, the degree of skin lesion recovery and rash elimination at 1 week and 1 month after treatment were compared. Compared with contrast group, patients in epidural infusion group had a remarkably higher degree of lesion recovery and herpes elimination at the two time points (*P* < 0.05; Tables 2, 3). The skin lesion recovery area at 1 month of contrast group was 79.33%, while that of group epidural infusion was significantly higher than that of contrast group (88.43%, *P* < 0.001). In addition, the rash elimination in group epidural infusion within 1 week was also significantly higher than that in contrast group (*P* < 0.001).

**Table 2:** Recovery of the skin lesion area (%).

**Time**	**Contrast group**	**Epidural infusion group**	***P*-value**
1 week	32.01 ± 15.07	37.30 ± 11.80	0.011
1 month	79.33 ± 12.45	88.43 ± 10.54	< 0.001

P-value, control group compared to the epidural infusion group.

**Table 3:** Elimination of the rash numbers (%).

**Time**	**Contrast group**	**Epidural infusion group**	***P*-value**
3 days	11.27 ± 7.88	21.27 ± 9.47	< 0.001
1 week	41.90 ± 24.12	62.06 ± 20.21	< 0.001
1 month	96.67 ± 8.26	98.76 ± 4.22	0.039

P-value, control group compared to the epidural infusion group.

**Figure 1:**
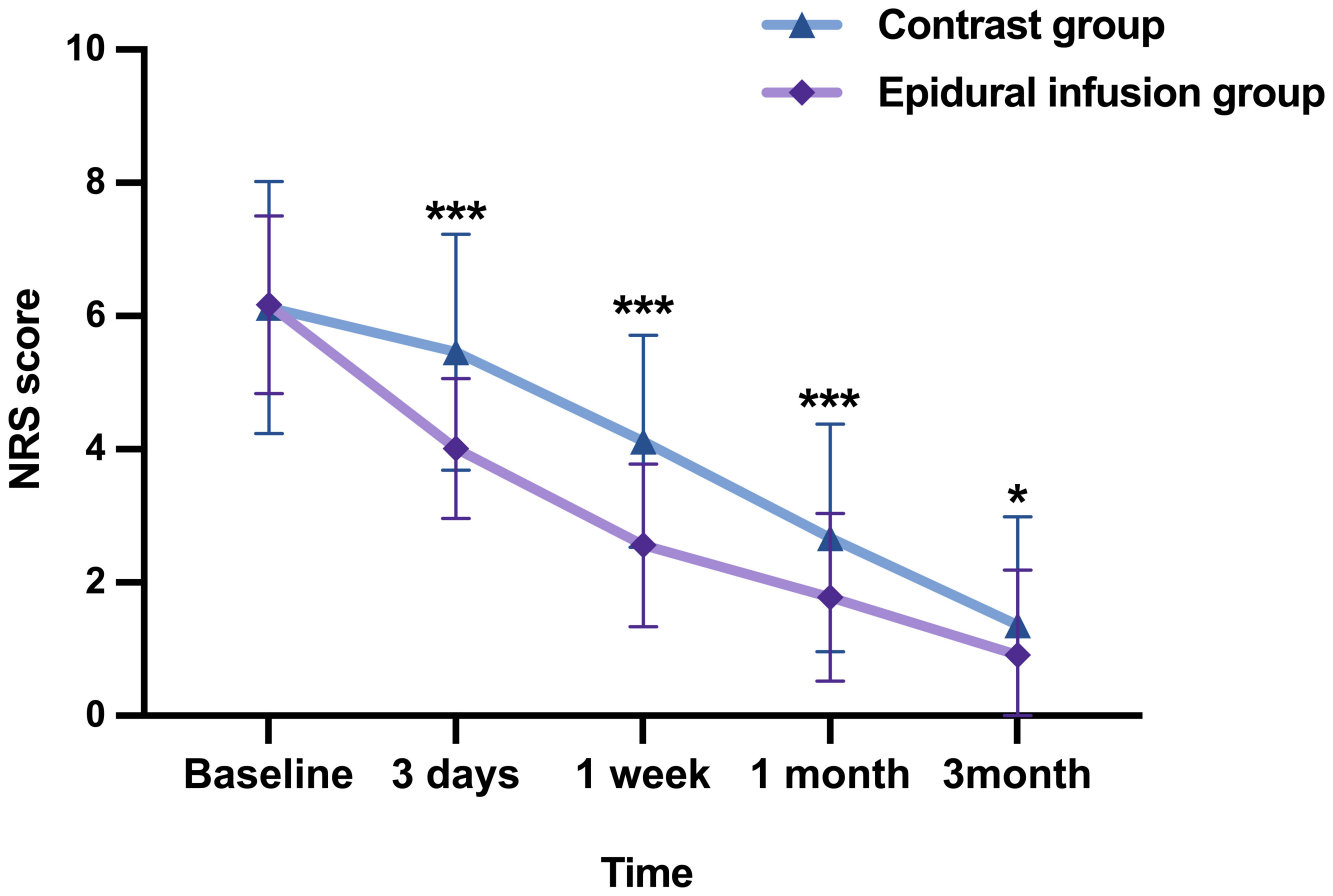
Overall change of NRS over time. NRS was reduced significantly at all time points compared to baseline. NRS, numerical rating scale. ***P < 0.001 compared to control group, *P < 0.05 compared to control group.

**Figure 2:**
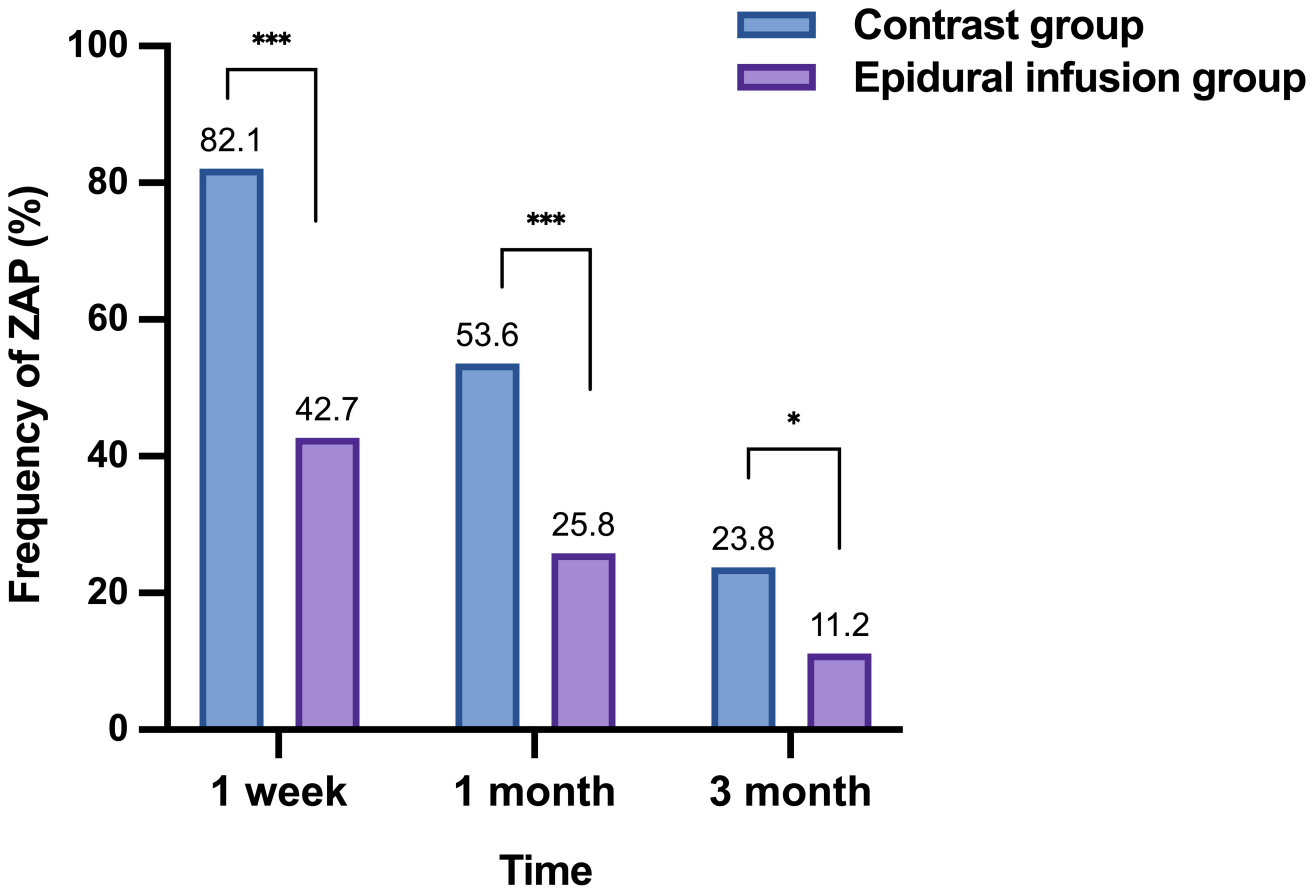
Frequency of ZAP over time. Compared with the control group, the epidural group had a significantly lower frequency of ZAP. ***P < 0.001 compared to control group, *P < 0.05 compared to control group.

The pain scores at different follow-up times after treatment in the two groups were compared to evaluate analgesic effects. NRS pain scores were lower after treatment than before treatment in both groups at all observation time points. There were significant differences in the post-treatment pain scores between the two groups after the 3 days and up to the 3rd months (*P* < 0.05). Specifically, patients in epidural infusion group displayed significantly lower pain scores than patients in contrast group at each follow-up time after treatment (*P* < 0.05; Table 4 and Figure 1). Additionally, fewer patients in epidural group reported ZAP than in contrast group at 1 month after treatment (OR = 0.30, 95% CI = 0.16–0.57, *P* < 0.001). The RD was −27.7%, meaning that nearly 3 out of 10 patients who received epidural injections would be free from ZAP within 1 month of epidural infusion compared with patients who receive oral medication only (Table 5 and Figure 2).

**Table 4:** Improvement of NRS score after treatment.

**NRS score**	**Contrast group**	**Epidural infusion group**	***P*-value**
Baseline	6.06 ± 1.97	5.98 ± 1.41	0.754
3 days	5.40 ± 1.80	4.35 ± 1.01	< 0.001
1 week	4.06 ± 1.60	3.01 ± 1.01	< 0.001
1 month	2.56 ± 1.72	1.94 ± 0.70	0.003
3 months	1.12 ± 1.40	0.75 ± 0.66	0.031

P-value, control group compared to the epidural infusion group.

**Table 5:** Proportion of patients with ZAP at different times (%).

**Group**	**1 week**	**1 month**	**3 months**
**Patients with ZAP**			
Contrast group	69/84 (82.1%)	45/84 (53.6%)	20/84 (23.8%)
Epidural infusion group	38/89 (42.7%)	23/89 (25.8%)	10/89 (11.2%)
*P-*value	< 0.001	< 0.001	0.028
**Risk**			
RD (95% CI)	−39.5% (0.25–0.54)	−27.7% (0.13–0.43)	−9.2% (−0.04–0.22)
OR (95% CI)	0.16 (0.08–0.33)	0.30 (0.16–0.57)	0.41 (0.18–0.93)

ZAP, zoster-associated pain (NRS ≥ 3); RD, risk difference; OR, odds ratio.

P-value, control group compared to the epidural infusion group.

**Figure 3:**
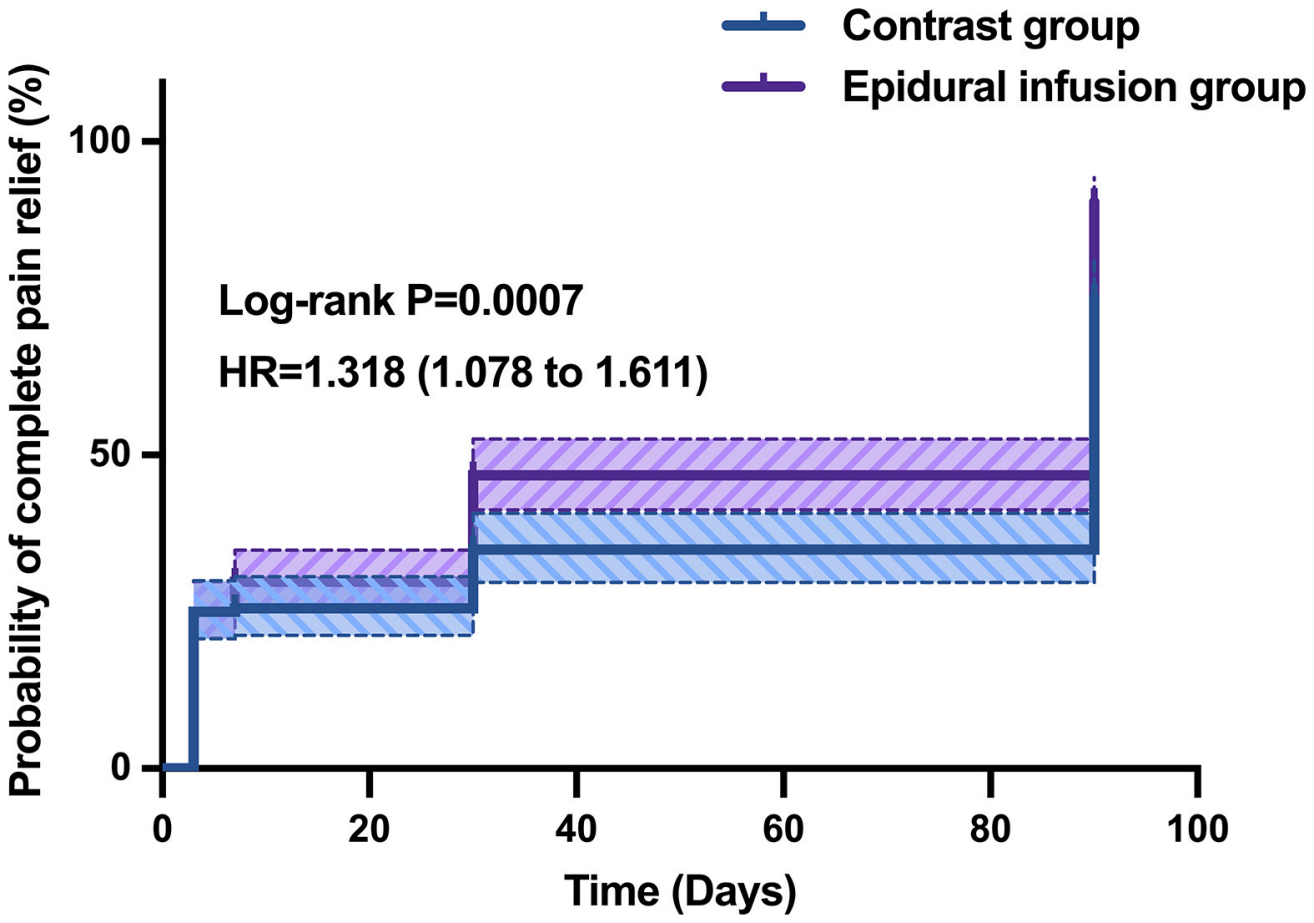
Kaplan–Meier curve of complete pain relief. The probability of complete pain relief of the epidural group was significantly higher.

Importantly, the information about pain relief in the 3rd month could contribute to evaluate the effect of the two therapies for the incidence of PHN. As seen in Table 5 and Figure 2, at the 3 months after treatment, the incidence of PHN was significantly reduced in epidural infusion group (*P* < 0.05), and 10 (11.2%) patients in epidural infusion group reported PHN, compared with 20 (23.8%) in contrast group advanced to PHN (OR = 0.41, 95% CI = 0.18–0.93, *P* = 0.028). The RD was −9.2%, indicating that epidural infusion was predicted to be effective in reducing the incidence of PHN compared with oral administration. The Breslow–Day test was used to assess the effect heterogeneity between different dermatomal regions at 1 month after treatment (χ^2^ = 1.74, df = 2, *P* = 0.419) and 3rd month (χ^2^ = 2.08, df = 2, *P* = 0.354), no significant differences were found. Therefore, the Mantel–Haenszel method was used to calculate the combined effect size. However, the subgroup data are provided for reference in Table 6. Furthermore, the Kaplan–Meier curve is shown in Figure 3. The log-rank tests revealed significant differences in the complete pain relief rates between the two groups (*P* = 0.0007, HR = 1.318, CI = 1.078–1.611; Figure 3). In 3 months after treatment, the percentage of patients with effective remission (≥50% reduction in pain severity) was not significantly different between groups (*P* = 0.540). However, the percentage of complete remission of PHN was significantly higher in epidural infusion group than in contrast group (82.0 vs. 61.9%, OR = 2.81, 95% CI = 1.40–5.64, *P* = 0.003; Table 7).

**Table 6:** Proportion of patients with ZAP at different times in different dermatomal locations (%).

**Dermatomal locations**	**Contrast group**	**Epidural infusion group**	**OR (95% CI)**	***P-*value**
**1 month**				
Cervical	50.0% (18/36)	36.8% (7/19)	0.79 (0.49, 1.27)	0.351
Thoracic	55.0% (22/40)	26.0% (13/50)	0.61 (0.42, 0.89)	0.005
Lumbar	62.5% (5/8)	15.0% (3/20)	0.44 (0.18, 1.10)	0.012
**3 months**				
Cervical	22.2% (8/36)	21.1% (4/19)	0.99 (0.74, 1.32)	0.920
Thoracic	25.0% (10/40)	10.0% (5/50)	0.83 (0.68, 1.02)	0.058
Lumbar	25.0% (2/8)	5.0% (1/20)	0.79 (0.52, 1.19)	0.122

ZAP, zoster-associated pain (NRS ≥ 3); OR, odds ratio.

P-value, control group compared to the epidural infusion group.

**Table 7:** Between-group comparisons of effective remission and complete remission in pain.

**Remission**	**Contrast group**	**Epidural infusion group**	**OR (95% CI)**	***P*-value**
**1 month**				
Effective remission	61/84 (72.6%)	75/89 (84.3%)	1.74 (0.96, 3.15)	0.061
Complete remission	21/84 (25.0%)	43/89 (48.3%)	1.45 (1.15, 1.84)	0.001
**3 months**				
Effective remission	73/84 (86.9%)	80/89 (89.9%)	1.30 (0.57–2.97)	0.540
Complete remission	52/84 (61.9%)	73/89 (82.0%)	2.81 (1.40–5.64)	0.003

OR, odds ratio.

P-value, control group compared to the epidural infusion group.

Complication rates (including nausea, vomiting, dysuria, itching sensation, and hypotension) during treatment were higher in contrast group than in epidural infusion group. However, these differences were not statistically significant (all *P* > 0.05; Table 8). Importantly, no serious surgery-related complications were detected in epidural infusion group. The risk-benefit ratios at 1 and 3 months after treatment were 13.7 and 11.8, respectively, both >1, indicating that the benefits outweigh the risks (Table 9).

**Table 8:** Comparison of complication rate.

**Side effect**	**Contrast group**	**Epidural infusion group**	***P*-value**
Nausea	4/84 (4.48%)	3/89 (3.37%)	0.642
Vomiting	3/84 (3.58%)	2/89 (2.25%)	0.603
Dysuria	0/84 (0.00%)	1/89 (1.12%)	0.248
Itching sensation	1/84 (1.20%)	0/89 (0.00%)	0.228
Hypotension	0/84 (0.00%)	1/89 (1.12%)	0.248

P-value, control group compared to the epidural infusion group.

**Table 9:** Risk-benefit ratio (RBR).

**Group**	**Complete remission rate**	**Complication rate**	**RBR**
**1 month**			
Contrast group	21/84 (25.0%)	8/84 (9.5%)	13.7
Epidural infusion group	43/89 (48.3%)	7/89 (7.8%)	
**3 months**			
Contrast group	52/84 (61.9%)	8/84 (9.5%)	11.8
Epidural infusion group	73/89 (82.0%)	7/89 (7.9%)	

RBR, Absolute benefit/ Absolute risk; Absolute benefit, Complete remission rate of contrast group/ Complete remission rate of epidural infusion group; Absolute risk, Complication rate of epidural infusion group/ complication rate of contrast group.

**Figure 4:**
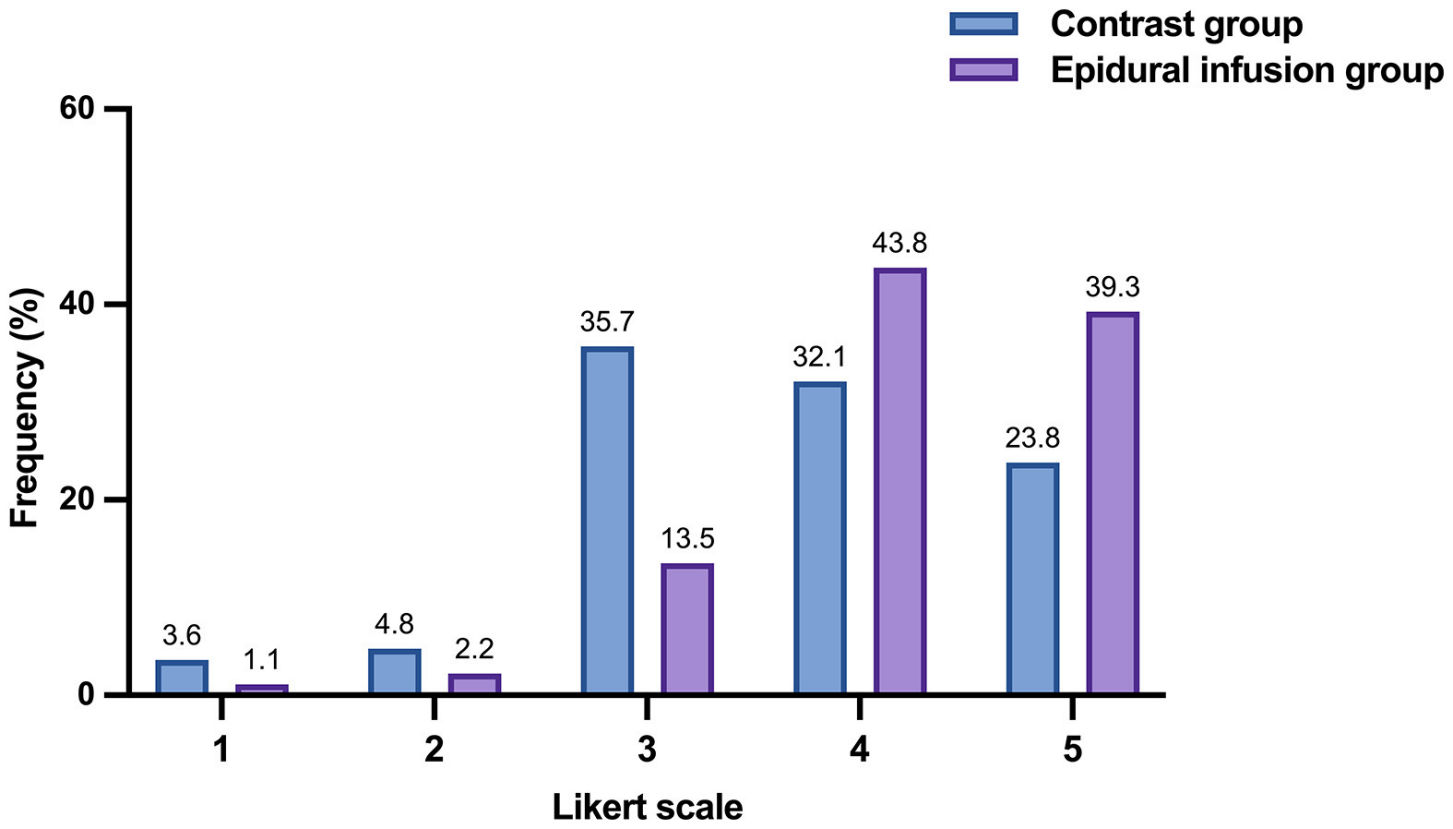
Distribution of the 5-point patient satisfaction scores in the epidural and control group. The epidural group has a higher Likert scale score.

Patient satisfaction scores in both groups were collected via 5-point Likert scale. There were remarkable differences between the two groups at 3 months after treatment. In terms of average satisfaction scores, the score of epidural infusion group was significantly higher than that of contrast group (4.18 ± 0.83 vs. 3.68 ± 1.01, *P* < 0.001; Table 10). In contrast to only 55.9% of patients in contrast group, 83.1% of patients in epidural infusion group showed Likert scale reached 4 points and above, which means the patients in epidural infusion group had higher satisfaction (Figure 4).

**Table 10:** Patient satisfaction (5-point Likert scale).

**Group**	**Likert scale**	***P*-value**
Contrast group	3.68 ± 1.01	< 0.001
Epidural infusion group	4.18 ± 0.83	

P-value, control group compared to the epidural infusion group.

## 4 Discussion

In this retrospective controlled trial, continuous epidural anesthetics and steroids infusion exhibited excellent therapeutic effect about skin lesions and rashes for acute HZ. Additionally, it could significantly reduce the incidence of PHN with its noteworthy analgesia for ZAP. During the follow-up of up to 3 months, pain remission manifested persistence accompanied by a few minor complications, demonstrating the effectiveness and safety.

The replication and transmission of the VZV in the skin and nerves lead to the major characteristics of HZ-related skin lesions and ZAP, and clinical symptoms appear in three stages: pre-eruptive, acute exsudative, and chronic stage (15–17). The skin lesions mainly include rashes, erythema, edema, and subsequent cicatrization. The representative rash and erythema generally appear after 2–3 days and lasts 3–5 days, affecting a single dermatome. Eventually, lesions usually heal gradually within 2–4 weeks after onset, followed by common scarring and pigmentation (18). HZ occurs worldwide without seasonal variations of incidence. The incidence increased with age, and that the severity increased sharply in those older than 60 years, ranging from 1.2 to 3.4/1,000 person years in young adults and 3.9–11.8/1,000 person years in elderly people (19, 20). Furthermore, as the population aging problem in China has entered a stage of rapid development, the number of HZ cases is expected to increase substantially. HZ has become a major public health problem, causing a huge burden on both health services and society. Therefore, finding a promising and ideal therapy is an urgent problem to be solved. As the critical complication of HZ, PHN has attracted attention due to its high incidence and severe clinical symptoms. Gauthier reported that 19.5% of herpes zoster patients develop PHN with pain persisting more than 1 month after HZ onset and 13.7% develop PHN with pain lasting at least 3 months (21). Previous studies have demonstrated that the incidence of PHN increases with age and affects women more than men (21–23). Owing to the long duration of pain, PHN has become a major concern that affects the quality of patients and needs to be treated seriously.

Although the pathophysiological mechanisms of acute HZ and PHN are not fully understood, HZ is considered to affect the skin and peripheral and central nervous systems, resulting in tissue inflammatory response. When the inflammatory response is intensified, it will lead to skin symptoms and ZAP through sensitization and de-afferentation (24, 25). Specifically, the loss of γ-aminobutyric acid inhibitory neurons and the drop of the nociceptor threshold induced by inflammatory mediators in the dorsal root ganglion (DRG) contribute to sensitization (26). And de-afferentation is resulted from dorsal root reorganization (24). Generally, since the above changes may not be established and prominent yet in acute HZ within 1 month after HZ onset, timely and proper inventions could alleviate the disease progression. Importantly, given that the dorsal root ganglion is a vital lesion site of HZ, it is reasonable and recommended to focus on the intervention of the dorsal root ganglion.

Due to the complexity of the pathogenesis of acute HZ and PHN, a great diversity of interventions have been proposed, including oral drug treatment (antiviral drugs, opioids, and corticosteroids), vaccinations, epidural injection, and other novel strategies, such as botulinum toxin type A injection (27), platelet-rich plasma injection (28), pulsed radio frequency (29), sympathetic nerve block (30), spinal cord stimulation (31), and so on. The above treatments have advantages and disadvantages: conventional oral administrations have some limitations and adverse reactions, and the emerging strategies are expensive and have technical barriers.

Steroids can attenuate the neurogenic inflammation and enhance the tissue repair via reducing edema and cytotoxicity (32, 33), has been reported as a treatment for HZ and PHN (34, 35). There are several routes of administration, and continuous epidural infusion was chosen in our study. Epidural administration of local anesthetics provides direct nerve blockade at the spinal level, leading to effective and sustained analgesia. The results showed that continuous epidural anesthetics and steroids infusion (epidural infusion group) could effectively control the skin lesions in acute stage. Compared with conventional oral medication management (contrast group), patients in epidural infusion group had a remarkably higher degree of lesion recovery and herpes elimination at 1 week and 1 month after treatment. Furthermore, NRS pain scores were lower after treatment than before and patients in epidural infusion group displayed significantly lower pain scores than patients in contrast group at each follow-up time after treatment. The underlying mechanisms of this beneficial therapeutic effects on skin lesions and pain in HZ are as follows: (1) drug: anti-inflammatory and anti-edema effects of steroids itself; local anesthetics provides direct nerve blockade, leading to effective and sustained analgesia; (2) drug administration: epidural administration targeting the affected spinal cord roots allows the drug to be applied directly to the area of the pathologic nerve, effectively controlling HZ symptoms; and (3) frequency: continuous low concentration administration allows the impaired nerve to be treated throughout the treatment process and avoids excessive hemodynamic changes owing to intervention, ensuring the continued efficacy and safety and reducing the occurrence of dose-related adverse reactions (36).

Although PHN has been defined as persistent pain after the healing of HZ, no consensus on time definition of PHN all over the world (37). Scholars generally believe that PHN is the pain lasting more than 3 months after the HZ onset (3, 38). However, the cutoff points for PHN diagnosis vary between 1 and 6 months in clinical. Therefore, in this study, incidence of ZAP at both 1 and 3 months were assessed, as both should be considered PHN from a clinical perspective. In order to evaluate the effect on the incidence of PHN, different perspectives were analyzed in this study. Firstly, fewer patients in epidural group reported ZAP than in contrast group at 1 and 3 months after treatment, and the risk difference was −27.7% after 1 month (meaning that nearly 3 out of 10 patients who received epidural injections would be free from ZAP within 1 month of epidural infusion compared with patients who receive oral medication only), and −9.2% after 3 months. Secondly, the proportion with different pain remission levels in 3 months after treatment were counted, the percentage of patients with effective remission was not significantly different between groups. However, the percentage of complete remission in 3 months was significantly higher in epidural infusion group than in contrast group. Taken together, the data indicated that a low possibility of transition from HZ to PHN if the continuous epidural anesthetics and steroids infusion was performed within the acute phase of HZ.

PHN in the head, face, and perineal regions responds poorly to epidural treatment, which is related to the complexity of nerve anatomy. Therefore, in this study, heterogeneity analysis of efficacy across different anatomical sites was conducted. Although no heterogeneity was found in treatment effects between different dermatomal regions, suggesting that the mechanism of epidural infusion may transcend local anatomical differences, there are also limitations. The Breslow–Day test is sensitive to sample size, and some subgroups (e.g., lumbar region, *n* = 45) may have small heterogeneity that was not detected. Subgroup analysis showed that, 1 month after treatment, the incidence of ZAP in the thoracic and lumbar regions was significantly reduced in the treatment group, suggesting that epidural infusion therapy may be more effective in the early treatment of thoracic and lumbar lesions.

The side effects of drugs have always been a major concern in clinical application. In order to evaluate safety, the incidences of complications in the two groups were explored in this study, and the results showed that there was no significant difference in the complication incidence between the two groups, and the safety of continuous epidural infusion was similar to that of oral administration. In addition, no serious surgery-related complications were detected in epidural infusion group. The risk-benefit ratios at 1 and 3 months after treatment indicated that the benefits of epidural anesthetics and steroids infusion therapy outweigh the risks. This targeted approach minimizes systemic side effects commonly associated with oral medication. It should be noted that all complications during the whole follow-up period were recorded to ensure the data integrity. These complications may be partly due to treatment, but may also be caused by other factors. Furthermore, the study also measured patients' subjective satisfaction via 5-point Likert scale. The data showed patients were clearly satisfied with this continuous epidural infusion therapy, and implied that this treatment had good acceptance and widespread would be possible.

There are several limitations to this research. Firstly, this study was not a prospective randomized controlled trial, and there may be an influence of unmeasured confounding variables. Secondly, to reduce the impact of other interventions on the results, the patients having a history of previous epidural injection or infusion treatment for HZ within 6 months were excluded. It may lead to the selection bias and information bias in the study. Thirdly, a relatively small sample size and data from a single center is another limitation of the present study. This study was retrospective dependent on the review of medical records, and it made it difficult to obtain an ideal sample size.

In conclusion, continuous epidural anesthetics and steroids infusion therapy may be effective in improving the symptoms of acute HZ and preventing the development of PHN. It can allow continuous administration of the drug to the exact site of the pathologic nerve to enhance the therapeutic effect. Due to the high safety and simple operation, this strategy is expected to be widely used in clinical practice. This study is an exploratory study, with conclusions requiring validation through further researches with a large sample and multi-center randomized controlled trials to strengthening the evidence for the superiority of continuous epidural anesthetics and steroids infusion in future.

## Data Availability

The raw data supporting the conclusions of this article will be made available by the authors, without undue reservation.

## References

[1] Schmader K. Herpes zoster. Ann Intern Med. (2018) 169:ITC19–31. 10.7326/L18-0558 30083718

[2] Rosamilia LL. Herpes zoster presentation, management, and prevention: a modern case-based review. Am J Clin Dermatol. (2020) 21:97–107. 10.1007/s40257-019-00483-1 31741185

[3] Kim HJ Ahn HS Lee JY Choi SS Cheong YS Kwon K. Effects of applying nerve blocks to prevent postherpetic neuralgia in patients with acute herpes zoster: a systematic review and meta-analysis. Korean J Pain. (2017) 30:3–17. 10.3344/kjp.2017.30.1.3 28119767 PMC5256258

[4] Werner RN Nikkels AF Marinovic B Schafer M Czarnecka-Operacz M Agius AM. European consensus-based (S2k) guideline on the management of herpes zoster - guided by the European Dermatology Forum (EDF) in cooperation with the European Academy of Dermatology and Venereology (EADV), part 1: diagnosis. J Eur Acad Dermatol Venereol. (2017) 31:9–19. 10.1111/jdv.13995 27804172

[5] Makharita MY Amr YM El-Bayoumy Y. Single paravertebral injection for acute thoracic herpes zoster: a randomized controlled trial. Pain Pract. (2015) 15:229–35. 10.1111/papr.12179 24528531

[6] Tang J Zhang Y Liu C Zeng A Song L. Therapeutic strategies for postherpetic neuralgia: mechanisms, treatments, and perspectives. Curr Pain Headache Rep. (2023) 27:307–19. 10.1007/s11916-023-01146-x 37493871

[7] Forbes HJ Thomas SL Smeeth L Clayton T Farmer R Bhaskaran K. A systematic review and meta-analysis of risk factors for postherpetic neuralgia. Pain. (2016) 157:30–54. 10.1097/j.pain.0000000000000307 26218719 PMC4685754

[8] Wei S Li X Wang H Liu Q Shao L. Analysis of the risk factors for postherpetic neuralgia. Dermatology. (2019) 235:426–33. 10.1159/000500482 31256167

[9] Saguil A Kane S Mercado M Lauters R. Herpes zoster and postherpetic neuralgia: prevention and management. Am Fam Physician. (2017) 96:656–63. 29431387

[10] Lin CS Lin YC Lao HC Chen CC. Interventional treatments for postherpetic neuralgia: a systematic review. Pain Physician. (2019) 22:209–28. 10.36076/ppj/2019.22.209 31151330

[11] Johnson RW McElhaney J. Postherpetic neuralgia in the elderly. Int J Clin Pract. (2009) 63:1386–91. 10.1111/j.1742-1241.2009.02089.x 19691624 PMC2779987

[12] Li SJ Feng D. Effect of 2% lidocaine continuous epidural infusion for thoracic or lumbar herpes-zoster-related pain. Medicine. (2018) 97:e11864. 10.1097/MD.0000000000011864 30095665 PMC6133574

[13] van Wijck AJ Opstelten W Moons KG van Essen GA Stolker RJ Kalkman CJ. The PINE study of epidural steroids and local anaesthetics to prevent postherpetic neuralgia: a randomised controlled trial. Lancet. (2006) 367:219–24. 10.1016/S0140-6736(06)68032-X 16427490

[14] Seo YG Kim SH Choi SS Lee MK Lee CH Kim JE. Effectiveness of continuous epidural analgesia on acute herpes zoster and postherpetic neuralgia: a retrospective study. Medicine. (2018) 97:e9837. 10.1097/MD.0000000000009837 29384888 PMC5805460

[15] Peng Q Guo X Luo Y Wang G Zhong L Zhu J. Dynamic immune landscape and VZV-specific T cell responses in patients with herpes zoster and postherpetic neuralgia. Front Immunol. (2022) 13:887892. 10.3389/fimmu.2022.887892 35720399 PMC9199063

[16] Zerboni L Sen N Oliver SL Arvin AM. Molecular mechanisms of varicella zoster virus pathogenesis. Nat Rev Microbiol. (2014) 12:197–210. 10.1038/nrmicro3215 24509782 PMC4066823

[17] Patil A Goldust M Wollina U. Herpes zoster: a review of clinical manifestations and management. Viruses. (2022) 14:192. 10.3390/v14020192 35215786 PMC8876683

[18] Cohen JI. Clinical practice: herpes zoster. N Engl J Med. (2013) 369:255–63. 10.1056/NEJMcp1302674 23863052 PMC4789101

[19] Zhang Z Liu X Suo L Zhao D Pan J Lu L. The incidence of herpes zoster in China: a meta-analysis and evidence quality assessment. Hum Vaccin Immunother. (2023) 19:2228169. 10.1080/21645515.2023.2228169 37424092 PMC10339760

[20] van Oorschot D Vroling H Bunge E Diaz-Decaro J Curran D Yawn B. Systematic literature review of herpes zoster incidence worldwide. Hum Vaccin Immunother. (2021) 17:1714–32. 10.1080/21645515.2020.1847582 33651654 PMC8115759

[21] Gauthier A Breuer J Carrington D Martin M Remy V. Epidemiology and cost of herpes zoster and post-herpetic neuralgia in the United Kingdom. Epidemiol Infect. (2009) 137:38–47. 10.1017/S0950268808000678 18466661

[22] Gialloreti LE Merito M Pezzotti P Naldi L Gatti A Beillat M. Epidemiology and economic burden of herpes zoster and post-herpetic neuralgia in Italy: a retrospective, population-based study. BMC Infect Dis. (2010) 10:230. 10.1186/1471-2334-10-230 20682044 PMC2921387

[23] Stein AN Britt H Harrison C Conway EL Cunningham A Macintyre CR. Herpes zoster burden of illness and health care resource utilisation in the Australian population aged 50 years and older. Vaccine. (2009) 27:520–9. 10.1016/j.vaccine.2008.11.012 19027048

[24] Apalla Z Sotiriou E Lallas A Lazaridou E Ioannides D. Botulinum toxin A in postherpetic neuralgia: a parallel, randomized, double-blind, single-dose, placebo-controlled trial. Clin J Pain. (2013) 29:857–64. 10.1097/AJP.0b013e31827a72d2 23370074

[25] Hu Y Zou L Qi X Lu Y Zhou X Mao Z. Subcutaneous botulinum toxin-A injection for treating postherpetic neuralgia. Dermatol Ther. (2020) 33:e13181. 10.1111/dth.13181 31769900

[26] Liu HT Tsai SK Kao MC Hu JS. Botulinum toxin A relieved neuropathic pain in a case of post-herpetic neuralgia. Pain Med. (2006) 7:89–91. 10.1111/j.1526-4637.2006.00100.x 16533208

[27] Intiso D Basciani M Santamato A Intiso M Di Rienzo F. Botulinum toxin type A for the treatment of neuropathic pain in neuro-rehabilitation. Toxins. (2015) 7:2454–80. 10.3390/toxins7072454 26134256 PMC4516923

[28] Zhou Z Hu X Yan F Zhou Y He R Ye X. Observation on the effect of platelet-rich plasma combined with drugs in the treatment of herpes zoster neuralgia. Int J Neurosci. (2024) 134:628–34. 10.1080/00207454.2022.2138381 36259487

[29] Ke M Yinghui F Yi J Xeuhua H Xiaoming L Zhijun C. Efficacy of pulsed radiofrequency in the treatment of thoracic postherpetic neuralgia from the angulus costae: a randomized, double-blinded, controlled trial. Pain Physician. (2013) 16:15–25. 10.36076/ppj.2013/16/15 23340530

[30] Abdelwahab EH Hodeib AA Marof HM Fattooh NH Afandy ME. Ultrasound-guided erector spinae block versus ultrasound-guided thoracic paravertebral block for pain relief in patients with acute thoracic herpes zoster: a randomized controlled trial. Pain Physician. (2022) 25:E977–85. 36288583

[31] Baek IY Park JY Kim HJ Yoon JU Byoen GJ Kim KH. Spinal cord stimulation in the treatment of postherpetic neuralgia in patients with chronic kidney disease: a case series and review of the literature. Korean J Pain. (2011) 24:154–7. 10.3344/kjp.2011.24.3.154 21935494 PMC3172329

[32] Dureja GP Usmani H Khan M Tahseen M Jamal A. Efficacy of intrathecal midazolam with or without epidural methylprednisolone for management of post-herpetic neuralgia involving lumbosacral dermatomes. Pain Physician. (2010) 13:213–21. 20495585

[33] Rijsdijk M van Wijck AJ Meulenhoff PC Kavelaars A van der Tweel I Kalkman CJ. No beneficial effect of intrathecal methylprednisolone acetate in postherpetic neuralgia patients. Eur J Pain. (2013) 17:714–23. 10.1002/j.1532-2149.2012.00233.x 23059790

[34] Cui JZ Zhang JW Yan F Yang XN Wang XL Zhao ZB. Effect of single intra-cutaneous injection for acute thoracic herpes zoster and incidence of postherpetic neuralgia. Pain Manag Nurs. (2018) 19:186–94. 10.1016/j.pmn.2017.09.002 29153295

[35] Makharita MY Amr YM. Effect of repeated paravertebral injections with local anesthetics and steroids on prevention of post-herpetic neuralgia. Pain Physician. (2020) 23:565–72. 10.36076/ppj.2020.23.565 33185373

[36] Rawal N. Epidural analgesia for postoperative pain: improving outcomes or adding risks? Best Pract Res Clin Anaesthesiol. (2021) 35:53–65. 10.1016/j.bpa.2020.12.001 33742578

[37] Pasqualucci A Pasqualucci V Galla F De Angelis V Marzocchi V Colussi R. Prevention of post-herpetic neuralgia: acyclovir and prednisolone versus epidural local anesthetic and methylprednisolone. Acta Anaesthesiol Scand. (2000) 44:910–8. 10.1034/j.1399-6576.2000.440803.x 10981565

[38] Kim YN Kim DW Kim ED. Efficacy of continuous epidural block in acute herpes zoster: incidence and predictive factors of postherpetic neuralgia, a retrospective single-center study. Medicine. (2016) 95:e4577. 10.1097/MD.0000000000004577 27512887 PMC4985342

